# Heat-Resistant Inbred Lines Coordinate the Heat Response Gene Expression Remarkably in Maize (*Zea mays* L.)

**DOI:** 10.3390/genes15030289

**Published:** 2024-02-25

**Authors:** Ming Xue, Xiaoyue Han, Luyao Zhang, Saihua Chen

**Affiliations:** 1 Jiangsu Key Laboratory of Crop Genomics and Molecular Breeding, Key Laboratory of Plant Functional Genomics of the Ministry of Education, Jiangsu Key Laboratory of Crop Genetics and Physiology, Agricultural College of Yangzhou University, Yangzhou 225009, Chinadx120210128@stu.yzu.edu.cn (L.Z.); 2 Jiangsu Co-Innovation Center for Modern Production Technology of Grain Crops, Yangzhou University, Yangzhou 225009, China

**Keywords:** maize, RNA-seq, heat resistant, heat response, genes

## Abstract

High temperatures are increasingly becoming a prominent environmental factor accelerating the adverse influence on the growth and development of maize (*Zea mays* L.). Therefore, it is critical to identify the key genes and pathways related to heat stress (HS) tolerance in maize. Great challenges have been faced in dissecting genetic mechanisms and uncovering master genes for HS tolerance. Here, Z58D showed more thermotolerance than AF171 at the seedling stage with a lower wilted leaf rate and H_2_O_2_ accumulation under HS conditions. Transcriptomic analysis identified 3006 differentially expressed genes (DEGs) in AF171 and 4273 DEGs in Z58D under HS treatments, respectively. Subsequently, GO enrichment analysis showed that commonly upregulated genes in AF171 and Z58D were significantly enriched in the following biological processes, including protein folding, response to heat, response to temperature stimulus and response to hydrogen peroxide. Moreover, the comparison between the two inbred lines under HS showed that response to heat and response to temperature stimulus were significantly over-represented for the 1234 upregulated genes in Z58D. Furthermore, more commonly upregulated genes exhibited higher expression levels in Z58D than AF171. In addition, maize inbred CIMBL55 was verified to be more tolerant than B73, and more commonly upregulated genes also showed higher expression levels in CIMBL55 than B73 under HS. These consistent results indicate that heat-resistant inbred lines may coordinate the remarkable expression of genes in order to recover from HS. Additionally, 35 DEGs were conserved among five inbred lines via comparative transcriptomic analysis. Most of them were more pronounced in Z58D than AF171 at the expression levels. These candidate genes may confer thermotolerance in maize.

## 1. Introduction

With global warming, temperatures beyond the physiological optimum for growth can induce heat stress (HS), which poses a threat to crop production worldwide [1]. High temperatures have become risks for abiotic stress factors that restrain crop development [2]. As has been reported, a mere 1 °C rise in the global mean temperature can result in an average reduction of 5.8% in global maize yields [3].

The optimal temperature range for maize growth is 25–33 °C during the day and 17–23 °C at night [4]. Warmer temperatures can accelerate the development rate, leading to less vegetative growth [5]. Further, increased temperatures during the flowering stages can harmfully impact the pollen vigor, anthesis–silking interval, pollination, grain filling and, ultimately, lead to lower yields [6,7,8,9]. However, high temperatures not only impact the reproductive growth stage but also affect the growth of maize seedlings. High temperatures can inhibit leaf photosynthesis and rubisco activation, resulting in increased thylakoid energization and transpiration rates. These effects can cause developmental delays or yield losses in maize [10].

Currently, a body of mechanisms have been discovered to regulate the heat tolerance of maize. For instance, HS can result in oxidative damage due to the excessive accumulation of reactive oxygen species (ROS) [11]. The level of ROS within plant cells is strictly controlled by the ROS scavenging system to maintain ROS homeostasis which has been found to be positively correlated with maize thermotolerance [12]. In addition, heat shock proteins (HSPs), known as molecular chaperones, participate in a wide range of biological processes including HS [13]. It has been shown that the overexpression of *HSP101* in anthers can enhance the capacity of heat tolerance in maize [14]. *ZmHsftf13*, which can rapidly induce the expression of *HSPs*, has been verified as positively regulating thermotolerance in the inbred line W22 [15]. Furthermore, the unfolded protein response (UPR) in different cellular compartments can be elicited by the accumulation of misfolded proteins under HS, which also contributes to the heat shock response in maize [16,17,18]. Additionally, plant hormones, such as abscisic acid (ABA), have versatile roles in regulating plant defense against temperature stress [19]. However, the mechanisms underlying why different maize inbred lines show the contrasting reaction under HS remain unclear. Therefore, it is critical to dissect and identify key components of HS tolerance in maize, which could facilitate the breeding of HS-tolerant varieties.

Transcriptomic analysis is a powerful means of identifying DEGs. Several functional genes have been characterized by it [20,21], and corn’s response to HS has been extensively studied using it over the past decades [15,18,22,23,24,25]. The genes involved in protein processing, the endoplasmic reticulum pathway, plant hormone signal transduction and other metabolic pathways may serve as critical components in response to HS [15,22]. However, as the most widely planted crop in the world, maize not only provides a large number of germplasm and genetic resources [26] but also presents great challenges in dissecting genetic mechanisms and exploring key genes related to HS tolerance. Therefore, it is of high importance to adopt more germplasm to uncover the genes associated with HS tolerance.

Zheng58 (Z58) is a highly esteemed elite inbred line used in maize breeding. It has been instrumental in the development of the maize hybrid ZD958, which is a popular summer maize variety that has been used in the Huang-Huai-Hai Plain over the past 20 years due to its HS resistance and other agronomic characteristics [27,28]. In the present research, an improved version of the Z58 inbred line, named Zheng58D (Z58D), and a heat-sensitive inbred line (AF171) were selected to dissect the mechanism underlying HS resistance and to identify the key heat-modulated genes in maize.

## 2. Materials and Methods

### 2.1. Plant Material and Growth Conditions

The maize inbred lines Z58D and AF171 (obtained from Yangzhou University, Yangzhou, China) were grown in a chamber (DARTH CARTER, Hefei, China; Catalog: RGL-P500-D3) under normal conditions (32 °C/22 °C, day/night) with a photoperiod of 16 h light and 8 h dark. The light intensities were ramped up to 1200 mmol/m^2^/s. Half of the plants at the two-leaf stage (V2, 10 days after seed germination) were cultivated under consistent conditions to ensure their growth and development, while others were moved to a higher temperature circumstance (42 °C/32 °C, day/night) for the HS treatment. The photoperiod and light intensities under the higher temperature conditions were set as the control condition. In order to avoid drought stress, the trays were adequately watered during the treatment. Samples were collected from the plants under the two conditions after 6 h of treatment for the mRNA-seq. Leaves under each condition were collected and stored at −80 °C after the evaporation of liquid nitrogen.

### 2.2. Histochemical Analyses

The accumulation of hydrogen peroxide (H_2_O_2_) was detected by 3,3′-diaminobenzidine (DAB) staining according to the supplier’s instructions (Nanjing Jiancheng Bioengineering Institute, Nanjing, China; Catalog: A064-1-1). The two inbred line plants were cultivated and subjected to HS and control conditions as mentioned above. Leaves in the same position were collected from each treatment for 78 h. Samples were then vacuum-infiltrated in DAB solution and incubated at 25 °C for 6 h. Samples were cleaned by boiling in ethanol before photography [29].

### 2.3. Bulk RNA Sequencing and Data Analysis

RNA was extracted from samples of leaf lamina obtained from the middle of the first fully expanded leaves. A total of 2 ug total RNA was used for the construction of sequencing libraries. Libraries for mRNA-seq were constructed and sequenced by Novogene Bioinformatics Technology Co., Ltd. (Beijing, China). Two biological replicates from each treatment were collected for both Z58D and AF171. Briefly, mRNAs were isolated from total RNAs using poly(A) selection. The isolated mRNAs were then fragmented into short fragments and reverse-transcribed to cDNAs. Illumina universal adapters were ligated and suitable fragments were selected for PCR amplification as templates. All the RNA-seq libraries were sequenced on Illumina platforms with PE150 strategy. mRNA sequencing data analysis was performed as reported previously [30]. After removing low-quality reads and illumina universal adapters, the remaining reads were then mapped to the reference genome (ZmB73_RefGen_v4, https://download.maizegdb.org/Zm-B73-REFERENCE-GRAMENE-4.0/, accessed on 10 June 2023) allowing one mismatch using STAR [31]. Gene expression levels were quantified as FPKM (fragments per kilobase of transcript per million fragments mapped). DESeq2 [32] was employed to identify DEGs between two samples which were named after the threshold of a false discovery rate (FDR) corrected *p* value < 0.01 and |log2 (fold-change)| > 1.The Gene Ontology (GO) enrichment analysis was conducted by the agriGO2 with the threshold of FDR < 0.01.

The RNA-seq data of CIMBL55 and B73 inbred line under 45 °C for 1 h and 6 h were employed to assess the global transcriptomic changes between heat-resistant and heat-sensitive inbred lines in response to HS [15]. In order to uncover the heat-responsive genes in maize seedlings, RNA-seq data of another inbred line, W22, under different temperature at V4 and V5 stage were also used [18].

### 2.4. RNA Extraction and RT-PCR Analysis

The total RNA of seedlings was extracted using the RNeasy Plant Mini kit (Vazyme, Nanjing, China) and the mRNA was reverse-transcribed into cDNA by the PrimeScript^TM^ Reverse Transcriptase Kit (Vazyme, Nanjing, China) following the manufacturer’s instructions. For qRT−qPCR analysis, the 2× SYBR green PCR master mix (Vazyme, Nanjing, China) was employed in conjunction with the ABI 7500 Real-Time PCR System (Applied Biosystems). The gene expression levels were quantified using the 2^−ΔΔCT^ method, with normalization against the control condition treatment. The maize *GAPDH* gene (*Zm00001d049641*) was employed as an internal control. Three biological replicates were used for gene expression analysis.

## 3. Results

### 3.1. Z58D Is more Tolerant Than AF171 under HS

**Figure 1:**
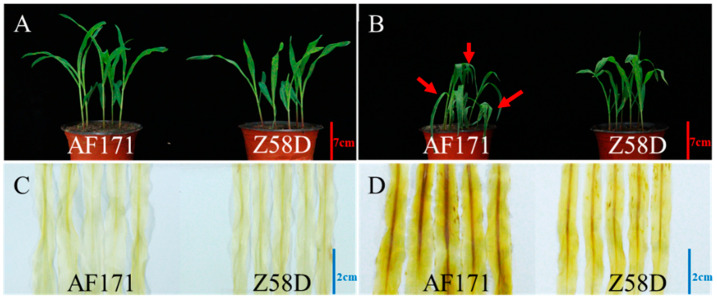
Phenotypes of AF171 and Z58D under different temperature conditions at the seedling stage. (**A**) The seedling phenotypes of AF171 and Z58D under normal conditions. (**B**) The seedling phenotypes of AF171 and Z58D subjected to heat stress (HS) treatment for 78 h. (**C**) DAB staining of AF171 and Z58D leaves under normal conditions. (**D**) DAB staining of AF171 and Z58D leaves subjected to HS treatment for 78 h. The red arrows indicate wilted leaves. Experiments were repeated three times with similar results.

To assess the thermotolerance of Z58D and AF171, the V2 seedlings were grown with or without HS treatments. There was no significant difference between Z58D and AF171 under normal conditions (Figure 1A). However, leaves of AF171 were severely wilted after 78 h HS treatment compared with Z58D (Figure 1B). H_2_O_2_ content assessment showed that more ROS accumulated in AF171 than Z58D after 78 h HS treatment detected by DAB staining (Figure 1C,D). These results indicated that Z58D was more thermotolerant than AF171.

### 3.2. Transcriptome Profiles of Seedlings from AF171 and Z58D under Normal and HS Conditions

To investigate the global transcriptomic changes for heat-sensitive and heat-resistant maize inbred lines at seedling stage in response to HS, we performed the comparison between AF171 and Z58D under HS (hereafter referred to as AF171−HS and Z58D−HS) and control conditions (hereafter referred to as AF171−CK and Z58D−CK) for 6 h by RNA-seq. In total, 1.9 billion high quality reads were obtained from eight cDNA libraries (Table S1). Principal component analysis revealed a distinct separation among different samples (Figure S1A). Furthermore, approximately 89.82% of the reads could be uniquely mapped to the B73 reference genome across all samples. Totally, 26,603 genes were expressed in at least one condition, whereas 14,495 genes were expressed in all the samples (Figure S1B, Table S2). These preliminary analyses with high reproducibility between the replicates and the different patterns of gene expression between inbred lines and conditions can fully support this study.

**Figure 2:**
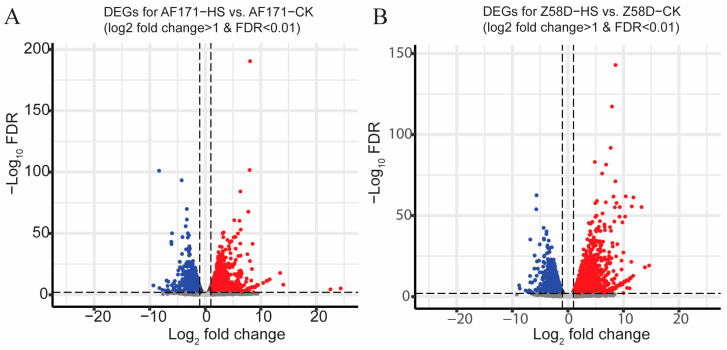
Volcano plot of differentially expressed genes (DEGs) in AF171 and Z58D by HS. (**A**) Volcano plot of DEGs for AF171−HS vs. AF171−CK. (**B**) Volcano plot of DEGs for Z58D−HS vs. Z58D−CK. *y*−axis showed statistical significance (−log_10_FDR). The *x*-axis showed fold-change in gene expression. Blue dots correspond to downregulated genes. Red dots correspond to upregulated genes.

The gene expression patterns were analyzed with the data. More DEGs were identified by comparison of the HS group and control group in Z58D than AF171. In total, 3006 and 4273 DEGs were identified in AF171 and Z58D, respectively (Figure 2A,B). There were 1722 upregulated genes and 1284 downregulated genes in AF171−HS vs. AF171−CK; meanwhile, there were 2426 upregulated genes and 1847 downregulated genes in Z58D−HS vs. Z58D−CK. Gene ontology (GO) analysis of the upregulated DEGs revealed that the top three enriched biological processes were ‘response to heat (GO:0009408), response to temperature stimulus (GO:0009266) and protein folding (GO:0006457)’ in AF171 and Z58D (Figure S2). In contrast, there was an existing obvious difference in the enriched pathways by downregulated DEGs. For instance, amide transport (GO:0042886), transmembrane transport (GO:0055085) and inorganic ion transmembrane transport (GO:0098660) were the top over-represented pathways in AF171 (Figure S2A), whereas ion transmembrane transport (GO:0034220), inorganic cation transmembrane transport (GO:0098662) and inorganic ion transmembrane transport occurred in Z58D (Figure S2B).

### 3.3. Commonly Regulated Genes in AF171 and Z58D by HS Treatment

**Figure 3:**
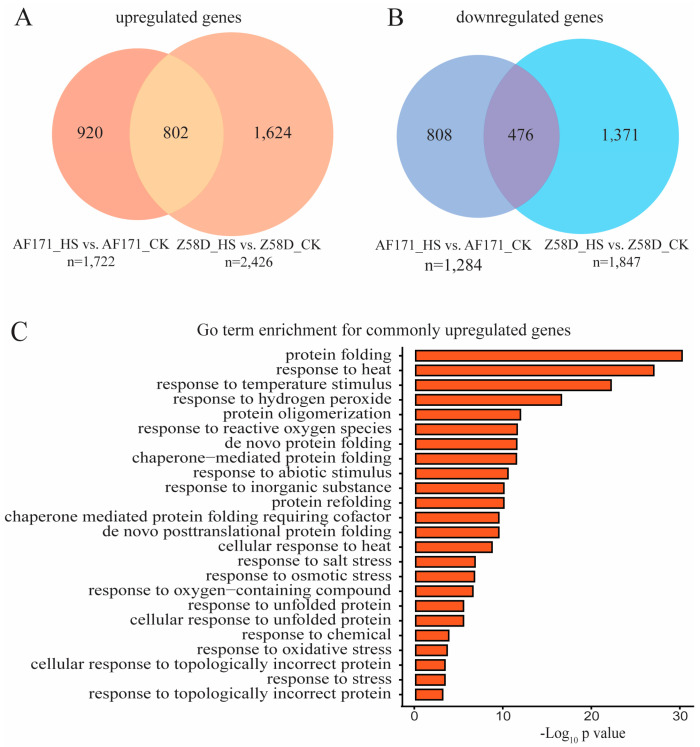
Heat-response DEGs in AF171 and Z58D seedlings. (**A**) Venn diagram summarizing the number of upregulated genes in AF171−HS vs. AF171−CK and Z58D−HS vs. Z58D−CK. (**B**) Venn diagram summarizing the numbers of downregulated genes in AF171−HS vs. AF171−CK and Z58D−HS vs. Z58D−CK. (**C**) Enriched biological process categories for the commonly upregulated DEGs in AF171−HS vs. AF171−CK and Z58D−HS vs. Z58D−CK.

A total of 802 genes were identified as commonly upregulated in both AF171−HS vs. AF171−CK and Z58D−HS vs. Z58D−CK (Figure 3A), whereas 476 genes were commonly downregulated (Figure 3B). For the commonly upregulated genes, GO analysis revealed that they were significantly assigned to the following biological processes, including protein folding, response to heat, response to temperature stimulus and response to hydrogen peroxide (GO:0009628) (Figure 3C). In contrast, no pathway was enriched for the commonly downregulated genes.

### 3.4. HS-Mediated Gene Expression Changes Remarkably in Heat-Resistant Inbred Lines

**Figure 4:**
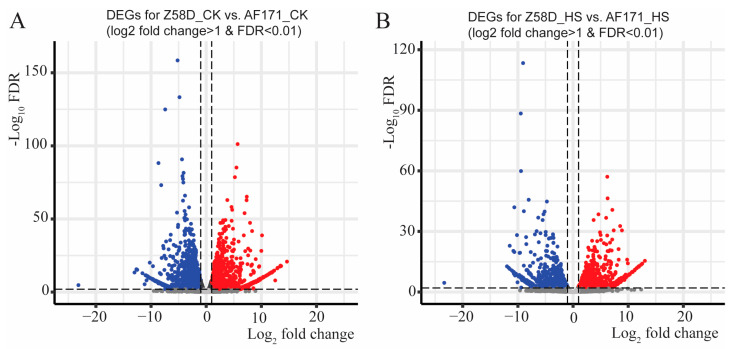
Volcano plot of DEGs between AF171 and Z58D under normal conditions and HS treatment. (**A**) Volcano plot of DEGs for Z58D−CK vs. AF171−CK. (**B**) Volcano plot of DEGs for Z58D−HS vs. AF171−HS. *y*-axis shows statistical significance (−log_10_FDR). *x*-axis shows fold-change in gene expression. Blue dots correspond to downregulated genes. Red dots correspond to upregulated genes.

**Figure 5:**
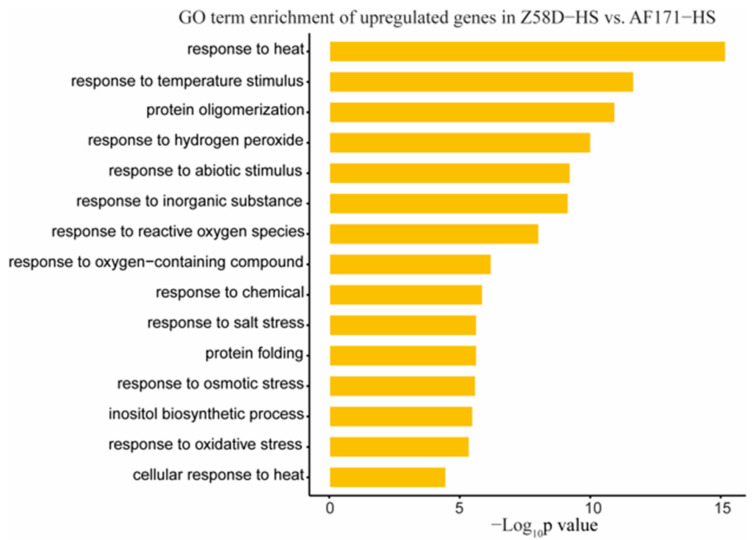
Enriched biological process categories of the upregulated genes in Z58D−HS vs. AF171−HS. Z58D−HS vs. AF171−HS refers to the comparison between Z58D and AF171 under HS. The x-axis represents minus log10 transformed *p* value.

To decipher the transcriptomic changes and the altered pathways in different inbred lines, 2969 DEGs were identified through the comparison of Z58D−CK and AF171−CK (Figure 4A). For the 1371 upregulated genes and 1598 downregulated genes, no significant biological processes showed up. Subsequently, we identified 1234 upregulated and 1214 downregulated genes in the comparison of Z58D−HS and AF171−HS (Figure 4B). The enriched GO terms differed notably from the observation under normal conditions. The upregulated genes were predominantly enriched in the pathways including response to heat and response to temperature stimulus (Figure 5), while no enriched pathways occurred for the downregulated genes.

**Figure 6:**
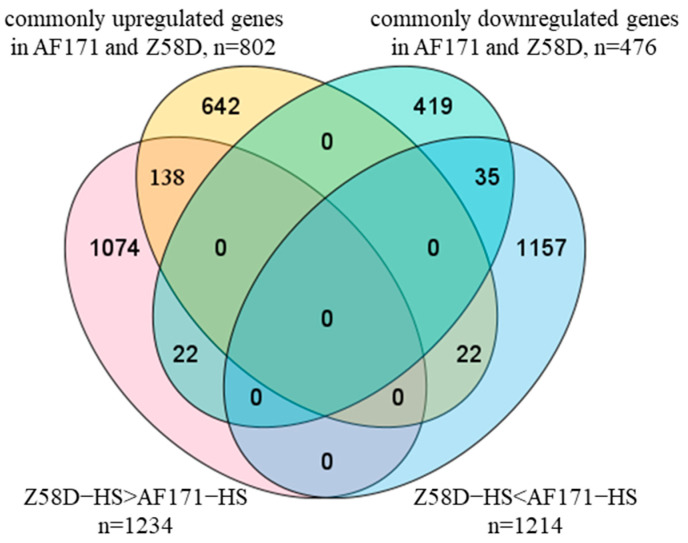
Number of overlapping and specific DEGs in AF171−HS vs. AF171−CK, Z58D−HS vs. Z58D−CK and Z58D−HS vs. AF171−HS. Yellow (commonly upregulated genes in AF171 and Z58D) represents the commonly upregulated genes in AF171−HS vs. AF171−CK and Z58D−HS vs. Z58D−CK. Green (commonly downregulated genes in AF171 and Z58D) represents the commonly downregulated genes in AF171−HS vs. AF171−CK and Z58D−HS vs. Z58D−CK. Pink (Z58D−HS > AF171−HS) represents DEGs with higher expression levels in Z58D than in AF171 under HS, blue (Z58D−HS < AF171−HS) represents DEGs with lower expression levels in Z58D than in AF171 under HS.

For the commonly upregulated genes in AF171−HS vs. AF171−CK and Z58D−HS vs. Z58D−CK, 138 genes (17.21%) were expressed at higher levels in Z58D than AF171 and 22 (2.74%) were expressed at lower levels in Z58D than AF171 (Figure 6). Overall, the HS-response gene expression changes were more dramatic in Z58D than AF171.

Moreover, RNA-seq data from B73 and CIMBL55 were employed and CIMBL55 was verified to be more tolerant than B73 under HS [15]. A total of 323 and 224 commonly upregulated genes in the two inbred lines after 1 h and 6 h HS treatment showed higher expression levels in CIMBL55 than B73, respectively. In contrast, only 41 and 111 commonly upregulated genes were expressed at lower levels in CIMBL55 than B73 after 1 h and 6 h under HS treatment, respectively (Figure S3). These results indicated that the HS-mediated gene expression changes were remarkable in heat-resistant inbred lines.

### 3.5. Heat-Resistant and Heat-Response Pathways and Gene Identification

To identify the heat-resistant pathways, we examined the specific upregulated genes in heat-resistant inbred lines. A total of 1624 specific upregulated genes were identified in Z58D−HS vs. Z58D−CK compared with AF171−HS vs. AF171−CK (Figure 3A and Figure S4A). Additionally, 2099 and 3663 specific upregulated genes were identified in CIMBL55 compared with B73 after 1 h and 6 h under HS treatment, respectively (Figure S4B,C). The GO analysis of the 1624 genes revealed that they were enriched in the response to abiotic stimulus (GO:0009628), temperature stimulus and heat (Figure S4D). For the 2099 genes, protein folding (GO:0006457) and de novo protein folding (GO:0006458) were significantly enriched. However, no significantly enriched pathways were identified for the 3663 genes (Figure S4D) and for the 274 specific upregulated genes in both Z58D and CIMBL55 (Figure S4E) with the threshold of FDR < 0.01.

**Figure 7:**
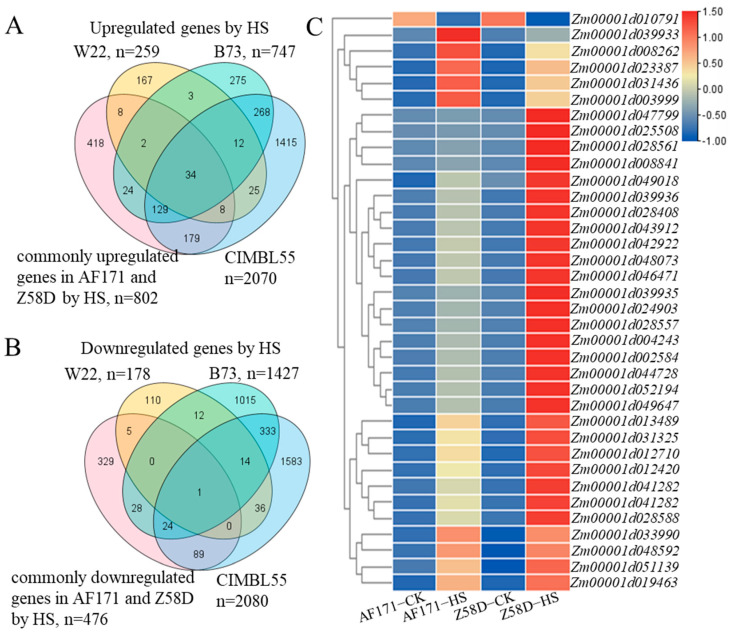
Specific and overlapping DEG numbers across five maize inbred lines. (**A**) Venn diagram summarizing the number of upregulated genes in five maize inbred lines. (**B**) Venn diagram summarizing the number of downregulated genes in five maize inbred lines. (**C**) Expression of 35 genes (overlapped DEGs in five maize inbred lines) in AF171 and Z58D under normal and HS conditions. The upregulated/downregulated (highlighted in yellow) gene numbers in W22 refer to these genes shared upregulated/downregulated in continuously increased heat-stress treatments (31 °C/21 °C, 33 °C/23 °C, 35 °C/25 °C and 37 °C/27 °C, day/night) at V4 or V5 leaves. The upregulated/downregulated gene numbers in CIMBL55 (highlighted in green) and B73 (highlighted in blue) refer to these genes shared upregulated/downregulated at 45 °C for 1 h and 6 h. Commonly upregulated/downregulated genes in AF171 and Z58D (highlighted in pink) represent the commonly upregulated genes in AF171−HS vs. AF171−CK and Z58D−HS vs. Z58D−CK.

In order to achieve a comprehensive understanding of heat-responsive genes in maize seedlings under HS, the RNA-seq data from W22, B73 and CIMBL55 were also analyzed [15,18]. Across all the datasets, 34 shared upregulated genes (Figure 7A) and 1 shared downregulated gene (Figure 7B) were identified. These candidate genes may contribute to heat tolerance in maize. Further analysis of these commonly expressed genes showed that the top enriched pathways were related to heat response, temperature stimulus response, hydrogen peroxide response and protein folding (Figure S5). Among the 34 upregulated genes, half of them belonged to the HSP genes, including 1 HSP90 gene, 5 HSP70 genes and 11 HSP20 genes (Table S3). The results indicated that the corn may evoke HSPs to cope with HS. Notably, these HS-mediated genes exhibited extremely different expression levels in the two inbred lines after HS treatment. They were more pronounced in Z58D (Figure 7C). Intriguingly, only one gene showed a significant reduction in all datasets. More studies are warranted to investigate its role in HS response.

### 3.6. Expression of the HSP, UPR Genes and Hydrogen Peroxide Gene under Different Temperature Conditions

**Figure 8:**
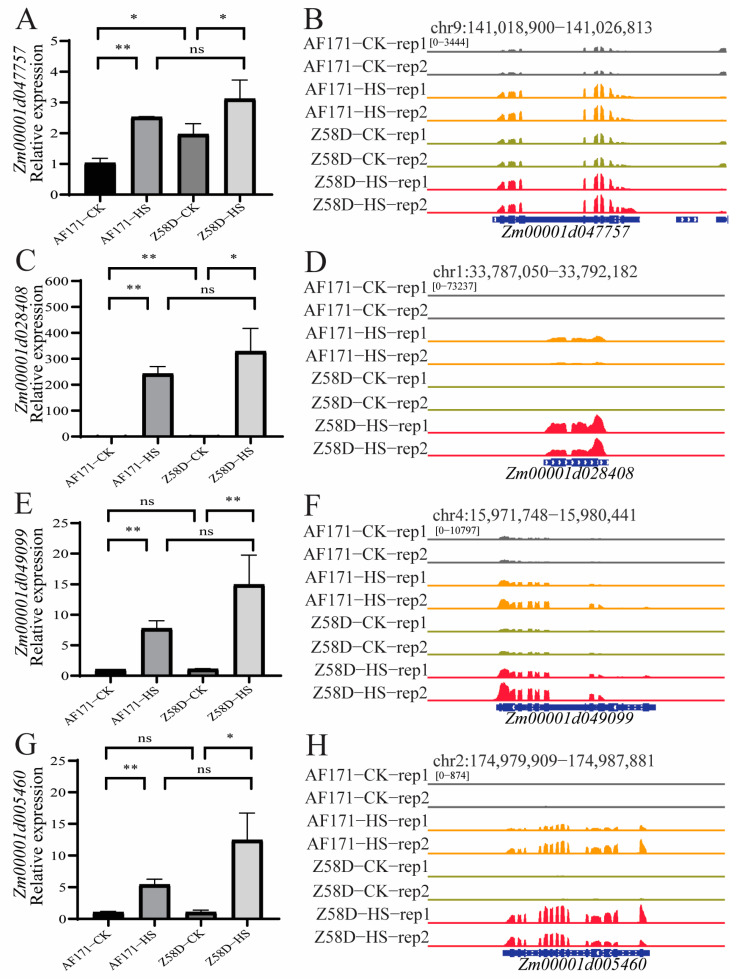
Relative expression pattern of four DEGs involved in three biological processes. (**A**,**C**,**E**,**G**): qRT−PCR analysis showing the relative expression *Zm00001d047757*, *Zm00001d028408*, *Zm00001d049099* and *Zm00001d005460*, respectively (* *p* < 0.05, ** *p* < 0.01, ns represents no significance). (**B**,**D**,**F**,**H**): snapshots of normalized RNA−seq at the locus as qRT−PCR quantified.

The upregulated genes in AF171−HS vs. AF171−CK, Z58D−HS vs. Z58D−CK and Z58D−HS vs. AF171−HS were most enriched in the biological processes of heat response, hydrogen peroxide and protein folding. We selected several genes which were associated with above-mentioned pathways under HS conditions and validated them through qRT-PCR, including one hydrogen peroxide gene (*Zm00001d047757*) [33] (Figure 8A,B), one HSP gene (*Zm00001d028408*) [18] (Figure 8C,D) and two canonical UPR genes (*Zm00001d049099* and *Zm00001d005460*) (Figure 8E,H) [18]. Primers are listed in Table S4. The trends in gene expression detected from qRT−PCR data were corroborated with IGV snapshots (Figure 8), which reflected that the RNA-seq data are reliable.

## 4. Discussion

With the rise in average global temperatures, the adverse effects of HS have exacerbated the impact on the growth and productivity of maize. The yield and quality of maize has been continually reduced over the past decades [27,28,34]. Therefore, it is crucial to elucidate the molecular mechanism of maize in response to HS.

In this study, heat-resistant (Z58D) and heat-sensitive (AF171) maize inbred lines were used for transcriptomic analysis. A total of 3006 DEGs in AF171 and 4273 DEGs in Z58D were identified, respectively. The results validated previous studies in CIMBL55 and B73, which also reported a huge number of DEGs in the heat-resistant inbred line [15]. The most enriched pathways for the upregulated genes in AF171 and Z58D were related to heat response, temperature stimulus response and protein folding, which confirmed the common mechanisms of maize in response to HS and showed the close link between the heat tolerance of maize and these pathways.

However, the underlying molecular mechanisms of the tolerance of different inbred lines to HS could show a significant variance. For instance, the enriched GO terms were different for the downregulated genes in AF171 and Z58D. Moreover, different results were also found in the GO term enrichment for Xiantian 5 and Zhefengtian 2 under HS, in which the top enriched terms related to “photosynthesis, oxidation-reduction process and photosynthesis, light reaction” for the upregulated genes and “translation, gene expression and cellular macromolecule biosynthetic process” for the downregulated genes [35]. In addition, the shared specific upregulated genes in heat-resistant inbred lines (Z58D and CIMBL55) do not enrich in any pathway (Figure S4). These different results observed could either be derived from the complicated genetic variations or from different HS treatments. The underlying mechanisms are still worth being uncovered.

Complicated processes were required to cope with heat stress in plants [36]. A single gene does not provide on its own the promising results required for developing heat-stress-tolerant varieties. However, some common pathways or DEGs may converge to improve heat stress tolerance in different maize inbred lines (Figure 3). In particular, HS-mediated gene expression changes seemed to be more significant in heat-resistant inbred lines than heat-sensitive inbred lines (Figure 6 and Figure S3). Namely, heat-resistant maize may coordinate a remarkable expression of genes in order to recover from HS. These results provide valuable insights for further investigation into the molecular mechanisms underlying the response and resistance to HS in maize.

By analyzing the DEGs of five maize inbred lines under HS conditions, thirty-five common DEGs were identified and most of the genes were related to either heat response or resistance. Among the 35 genes, 17 genes belonged to the HSP gene family which has been proved to function in plant tolerance to HS [37]. Apart from the HSP genes, other genes may also play important roles in heat resistance in maize (Figure S6). For instance, *Zm00001d049018* is a homologous gene of *AtPARK13* which encodes for a mitochondrial protease and has been ascertained as being induced by HS and conferring thermotolerance by degrading misfolded proteins in Arabidopsis [38]. *Zm00001d046471* is similar to *ATMBF1c* which is a heat response regulon. The DNA-binding domain of MBF1c has a dominant-negative effect on heat tolerance when constitutively expressed, whereas the ectopic expression of *MBF1c* enhances tolerance to heat in Arabidopsis [39,40]. The overexpression of wheat *MBF1c* can also confer heat tolerance in both yeast and rice [41]. Furthermore, the overexpression of *ROF1*, which is homologous to *Zm00001d004243*, can improve survival more than wild type plants when exposed to 45 °C in Arabidopsis. Further investigations have revealed that *ROF1* can enhance thermotolerance by interacting with HSP90.1 and influencing the accumulation of HsfA2-regulated small HSPs [42]. Additionally, alternative splicing plays a key role in heat-stress responses [43]. The step II splicing factor AtPRP18a (homologous to Zm00001d013489) has been identified as binding with nuclear cyclophilin18-1 which is crucial for the efficient splicing of retained introns and rapid responses to HS in Arabidopsis [44,45]. Finally, *AT1G30070* and *AT1G03070* (homologous to *Zm00001d012710* and *Zm00001d033990*, respectively) were also upregulated by HS treatment in Arabidopsis [46]. These genes may play crucial roles functionally in enhancing heat resistance in maize and can be used for heat resistant breeding.

## 5. Conclusions

HS is threating the yield and quality of maize on a global scale. However, the underlying mechanisms response and resistance to HS still need to be elucidated. In this study, we observed that Z58D was more thermotolerant than AF171 with a lower wilted leaf rate and H_2_O_2_ accumulation under HS conditions. A total of 3006 and 4273 DEGs were identified in AF171 and Z58D, respectively. The top three enriched biological pathways among the upregulated DEGs were consistent between the two varieties. However, significant differences were observed in the GO terms for the downregulated DEGs. Notably, 802 commonly upregulated genes in AF171 and Z58D were most enriched in the pathways related to protein folding, heat response, temperature stimulus response and hydrogen peroxide response. Furthermore, a comparative analysis revealed 2969 DEGs between AF171 and Z58D under normal conditions and 2448 DEGs under HS conditions. The upregulated genes in Z58D compared to AF171 under HS conditions were predominantly enriched in the pathway related to heat and temperature stimulus response. Additionally, 138 commonly upregulated genes in AF171 and Z58D exhibited higher expression levels in Z58D than AF171 and 22 genes expressed at lower levels in Z58D. Similar trends were observed in the comparison between CIMBL55 and B73 with genes showing a higher expression in CIMBL55 under heat stress conditions. A total of 35 commonly regulated genes overlapped in five maize inbred lines and several of them were validated through qRT-PCR. These findings suggest that these genes may hold the potential for enhancing heat resistance in maize breeding programs.

## Data Availability

All data supporting reported materials are available in the manuscript and Supplementary Materials. Bulk RNA-seq data in this study are already deposited in GEO with accession number: GSE254852.

## Supplementary Materials

The following supporting information can be downloaded at: https://www.mdpi.com/article/10.3390/genes15030289/s1. Table S1, summary of reads mapping to the reference genome; Table S2, raw data for FPKM values for all mRNA identified in AF171 and Z58D under different treatments; Table S3, annotation of the commonly regulated genes in five maize inbred lines under HS; Table S4, primers used in this study; Figure S1, gene expression patterns in AF171 and Z58D under different temperature conditions; Figure S2, gene ontology (GO) analysis of differently expressed genes (DEGs) in AF171 and Z58D by HS; Figure S3, numbers of overlapping and specific DEGs in CIMBL55, B73 and CIMBL55 vs. B73 under HS; Figure S4, specific upregulated genes in heat-resistant inbred lines; Figure S5, GO term enrichment for the commonly upregulated genes by heat stress across five maize inbred lines; Figure S6, genome browser snapshot of six upregulated genes in five inbred lines by heat stress.
