# Supplementary material for: Heat-Resistant Inbred Lines Coordinate the Heat Response Gene Expression Remarkably in Maize (Zea mays L.)
Source: Genes (Basel). 2024 Feb 25;15(3):289. doi: 10.3390/genes15030289 (PMC10970198; doi:10.3390/genes15030289)
Supplement: Supplementary file 1 [file genes-15-00289-s001.zip › Supplemental figures.pdf]

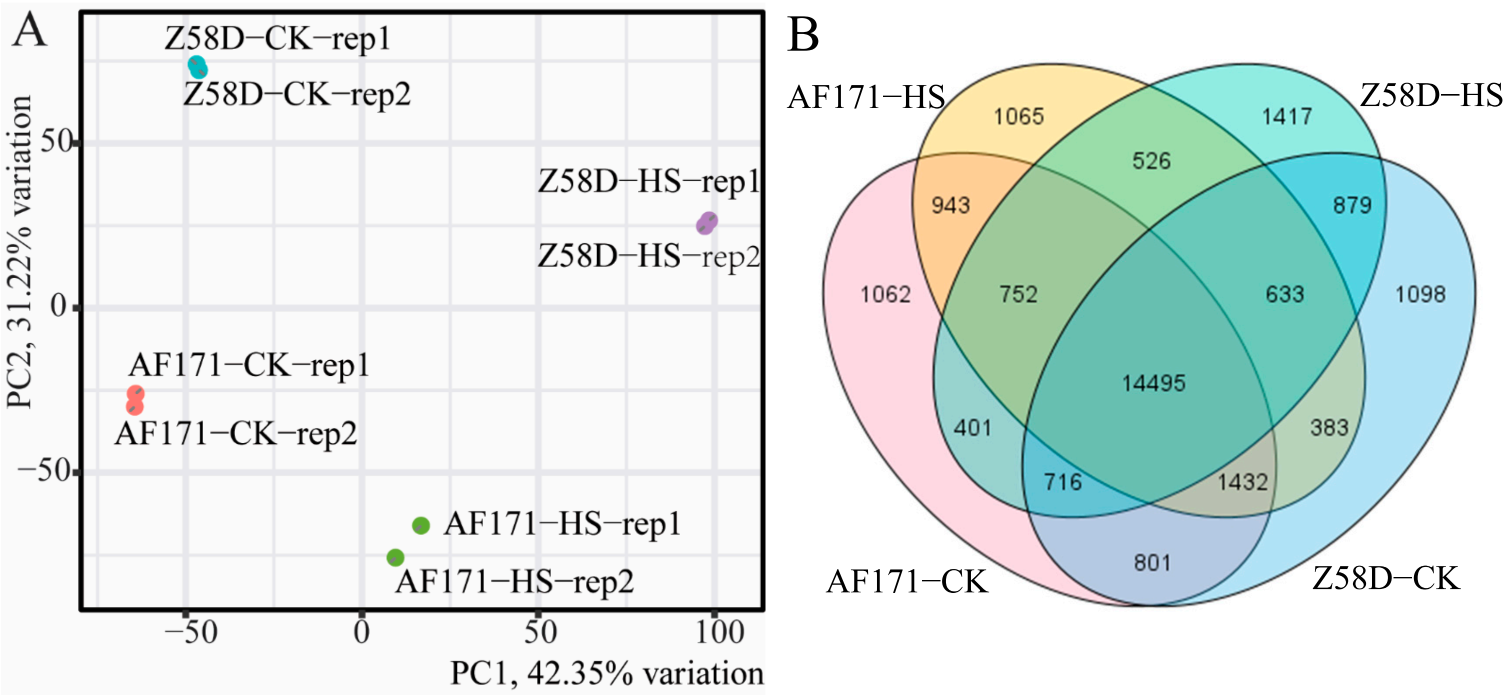

Figure S1. Gene expression patterns in AF171 and Z58D under different temperature conditions. (A) Principal component analysis (PCA) plots of eight libraries. (B) Number of expressed genes in AF171 and Z58D under different temperature conditions.

A

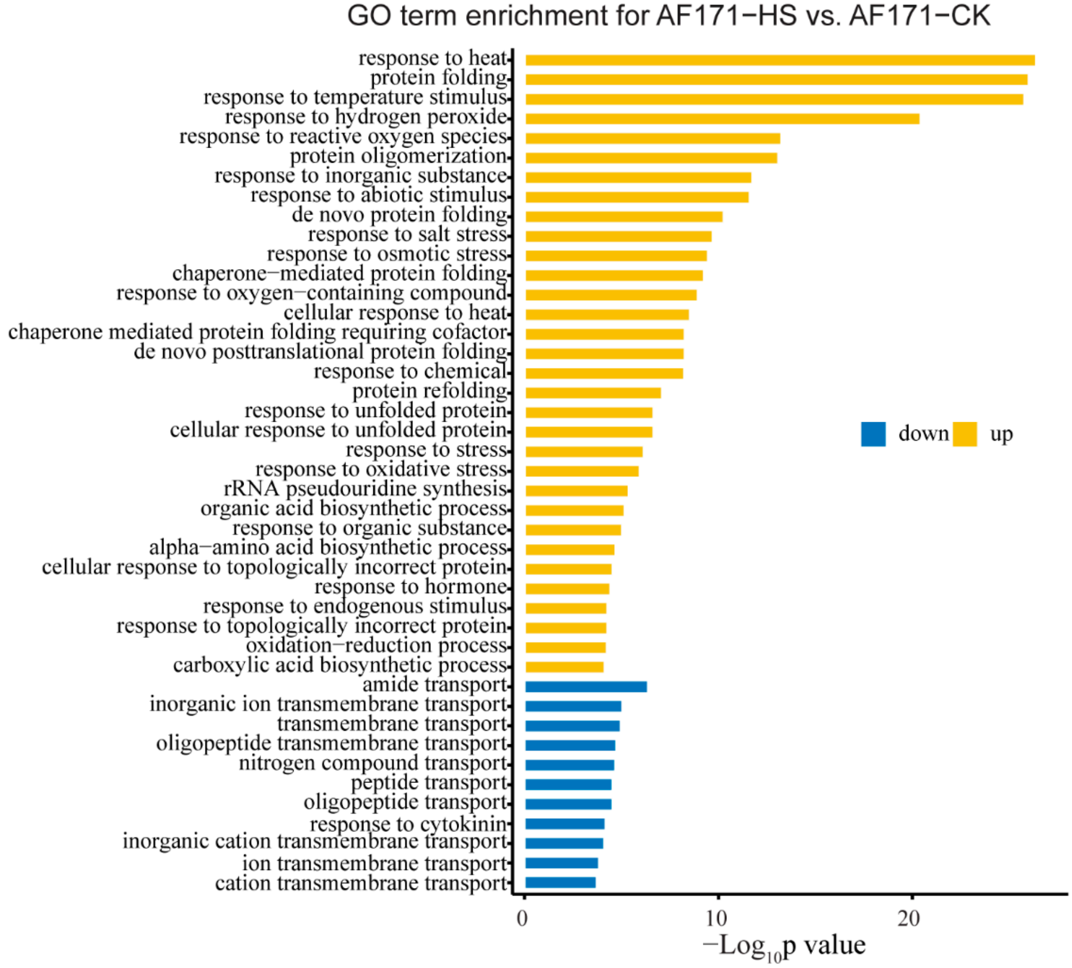

B

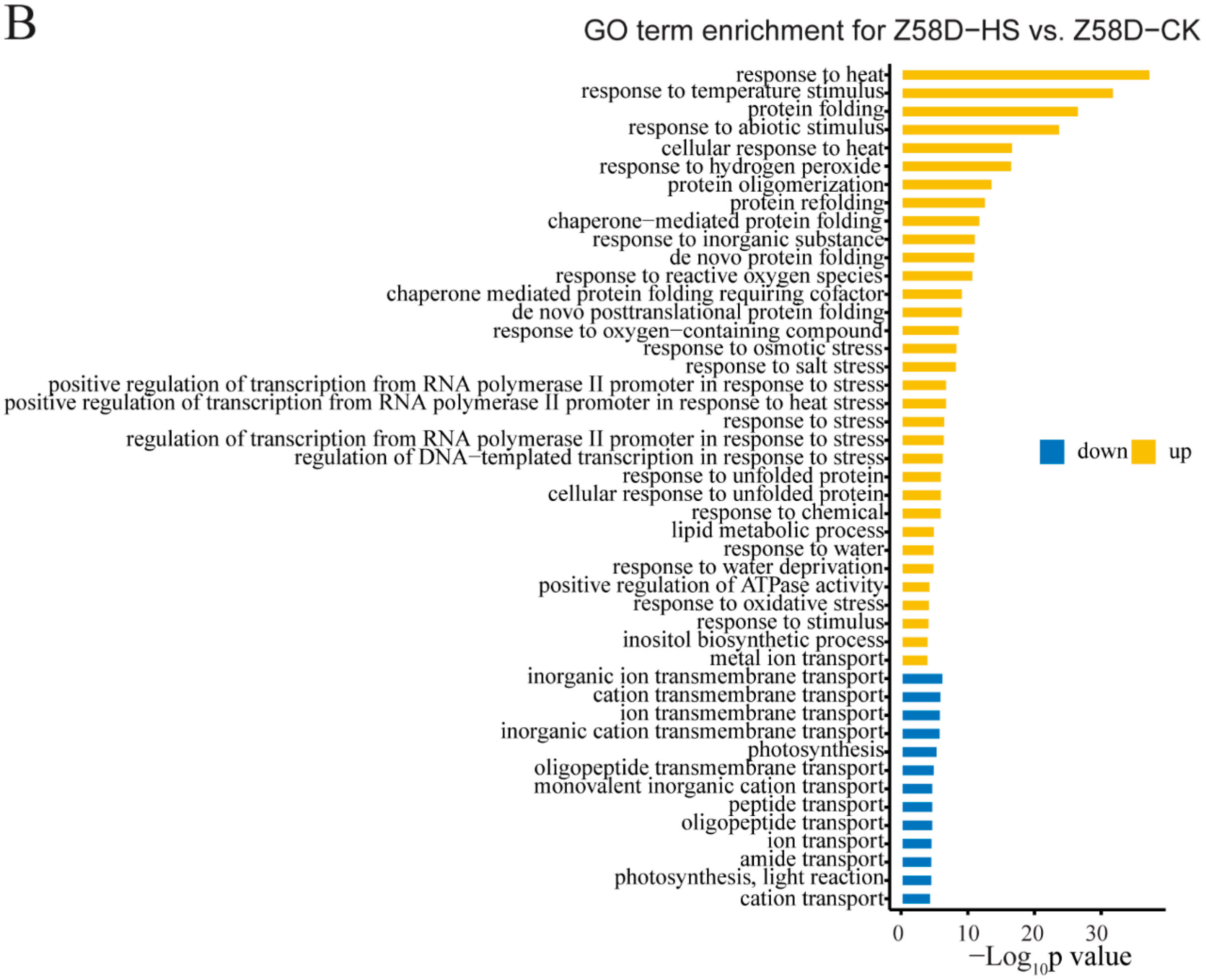

Figure S2. Gene ontology (GO) analysis of differently expressed genes (DEGs) in AF171 and Z58D by HS. (A) GO analysis of DEGs in AF171 by HS. (B) GO analysis of DEGs in Z58D by HS. The x-axis represents minus log10 transformed p value. Blue means the enriched pathway by using downregulated genes. Orange means the enriched pathway by using upregulated genes.

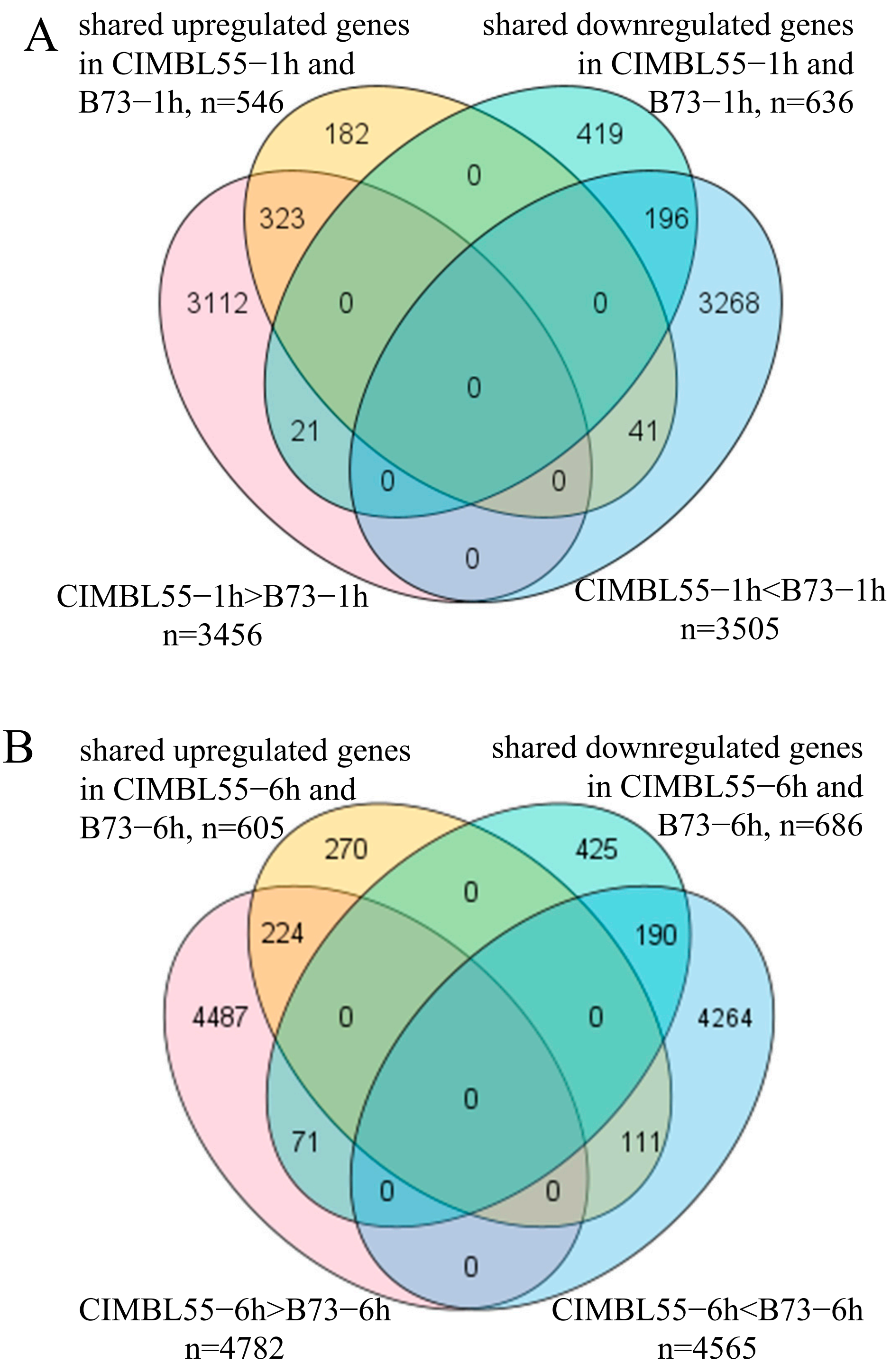

Figure S3. Numbers of overlapping and specific DEGs in CIMBL55, B73 and CIMBL55 vs B73 under HS. (A) Numbers of specific and overlapping DEGs in the four comparisons after 1 h HS treatment (referred to as CIMBL55−1h and B73−1h). Shared upregulated/downregulated genes (highlighted in yellow/green) in CIMBL55−1h and B73−1h represent these genes shared upregulated/downregulated in CIMBL55−1h vs. CIMBL55−0h and B73−1h vs. B73−0h. (B) Numbers of specific and overlapping DEGs in the four comparisons after 6 h HS treatment (referred to as CIMBL55−6h and B73−6h). Shared upregulated/downregulated genes (highlighted in yellow/green) in CIMBL55−6h and B73−6h represent these genes shared upregulated/downregulated in CIMBL55−6h vs. CIMBL55−0h and B73−6h vs. B73−6h. CIMBL55−1h> B73−1h and CIMBL55−6h>B73−6h for DEGs (highlighted in pink) with higher expression levels in CIMBL55 than B73 after 1 h and 6 h HS treatment, respectively. CIMBL55−1h<B73−1h and CIMBL55−6h<B73−6h for DEGs (highlighted in blue) with lower expression levels in CIMBL55 than B73 after 1 h and 6 h HS treatment, respectively.

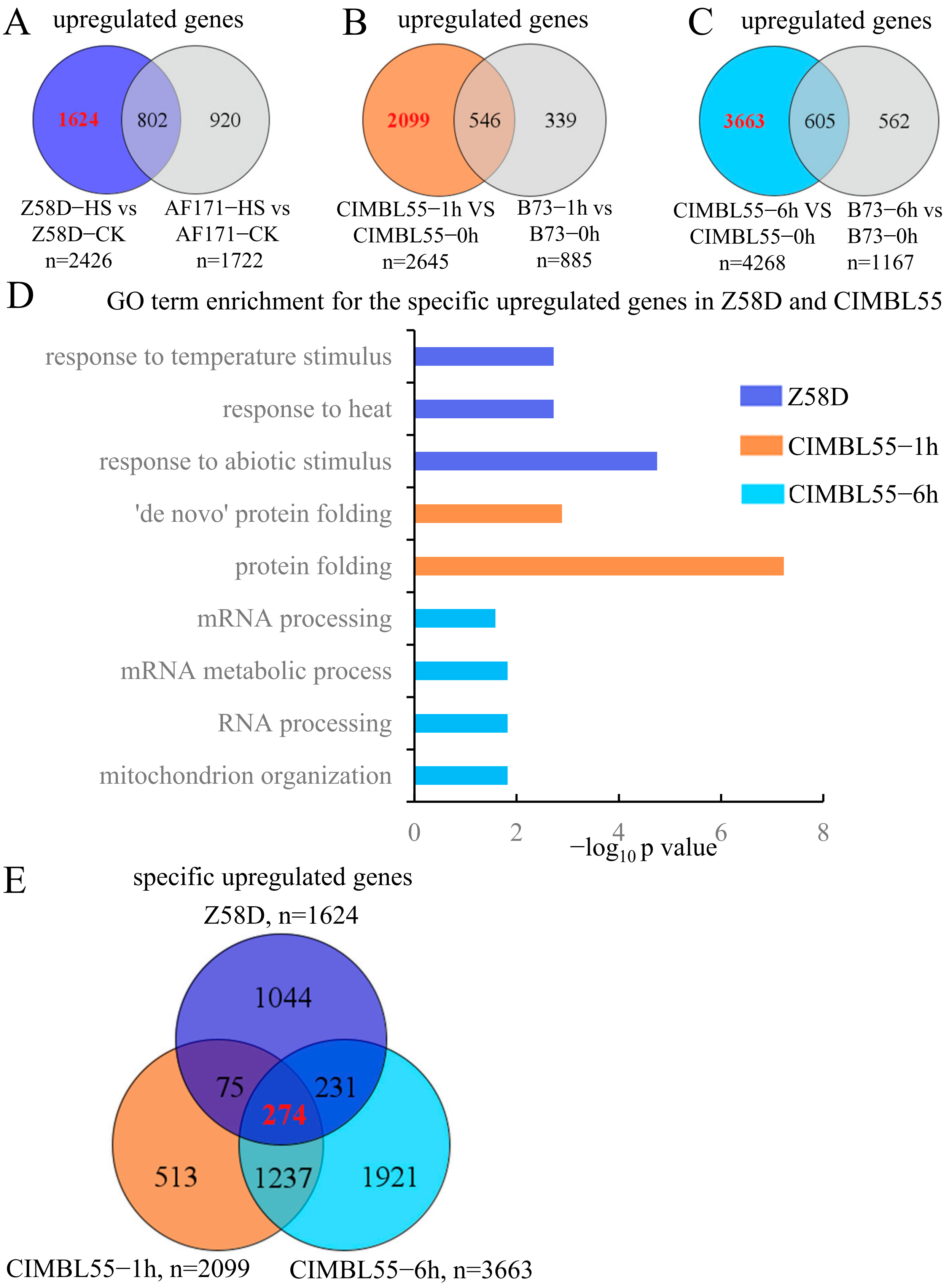

Figure S4. Specific upregulated genes in heat−resistant inbred lines. (A) Venn diagram summarizing the numbers of upregulated genes in AF171−HS vs. AF171−CK and Z58D−HS vs. Z58D−CK . (B) Venn diagram summarizing the numbers of upregulated genes in CIMBL55−1h vs. CIMBL55−0h and B73−1h vs. B73−0h. (C) Venn diagram summarizing the numbers of upregulated genes in CIMBL55−6h vs. CIMBL55−0h and B73−6h vs. B73−6h. (D) GO term enrichment for the specific upregulated genes in Z58D compared with AF171 or CIMBL55 compared with B73. (E) Venn diagram summarizing the numbers of the specific upregulated genes in Z58D and CIMBL55. Blue represents these genes specific upregulated in Z58D−HS vs Z58D−CK compared with AF171−HS vs AF171−CK. Orange represents these genes specific upregulated in CIMBL55−1h vs. CIMBL−0h compared with B73−1h vs. B73−0h. Cyan represents these genes specific upregulated in CIMBL55−6h vs. CIMBL−0h compared with B73−6h vs. B73−0h.

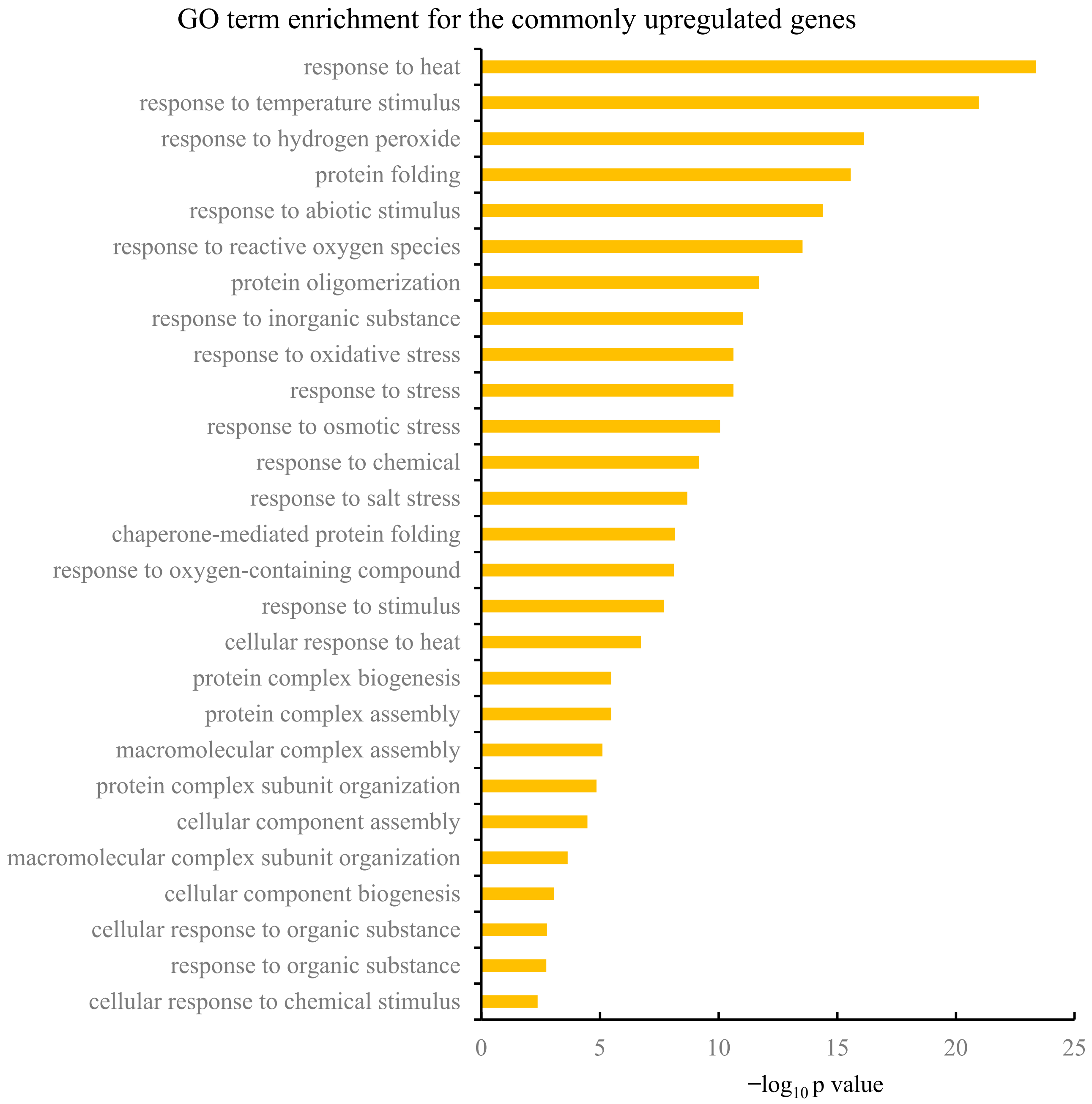

Figure S5. Enriched biological process categories of the commonly upregulated genes by heat stress across five maize inbred lines. The x-axis represents minus log10 transformed p value.

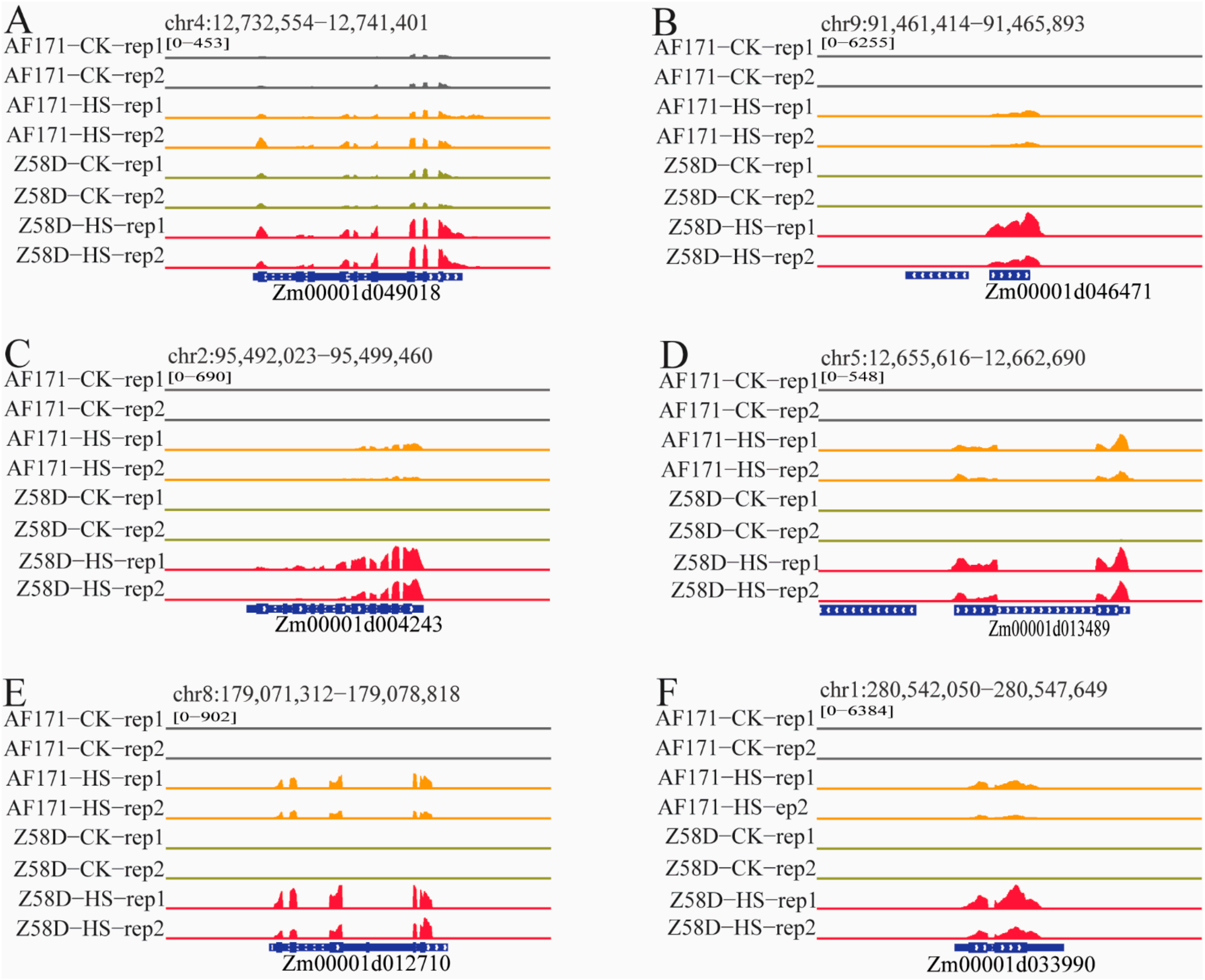

Figure S6. Genome browser snapshot of six upregulated genes in five inbred lines by heat stress.
